# Weak stromal Caveolin-1 expression in colorectal liver metastases predicts poor prognosis after hepatectomy for liver-only colorectal metastases

**DOI:** 10.1038/s41598-017-02251-9

**Published:** 2017-05-17

**Authors:** Kyriakos Neofytou, Emmanouil Pikoulis, Athanasios Petrou, Georgios Agrogiannis, Christos Petrides, Ioannis Papakonstandinou, Alexandros Papalambros, Anastasios Aggelou, Nikolaos Kavatzas, Theodoros Liakakos, Evangelos Felekouras

**Affiliations:** 1 Americam Medical Center, Nicosia, Cyprus; 2 University of Athens Medical School, Laikon Teaching Hospital, First Department of Surgery, Athens, Greece; 3 Nicosia General Hospital, Nicosia Surgical Department, Division of Hepatobiliary Pancreatic Surgery, Nicosia, Cyprus; 4 University of Athens Medical School, First Department of Pathology, Athens, Greece; 5 University of Athens Medical School, Areteio Teaching Hospital, Department of Surgery, Athens, Greece

## Abstract

Loss of stromal Caveolin-1 (CAV1) expression is associated with poor prognosis in various cancers. We evaluated the prognostic value of CAV1 expression of both cancer cells and stromal cells in colorectal liver metastases (CRLM) in patients undergoing hepatectomy. In this retrospective study, 109 patients were enrolled. CAV1 expression was studied by immunohistochemistry. The staining was scored semiquantitatively as weak or strong. Disease-free survival (DFS) and overall survival (OS) were calculated using both Kaplan–Meier and multivariate Coxregression methods. Weak stromal CAV1 expression was associated with decreased DFS and OS in univariate and in multivariate analysis (HR 2.00; 95% CI, 1.24–3.22; *P* = 0.004, and HR 2.47; 95% CI, 1.28–4.76; *P* = 0.007, respectively). Cancer cell CAV1 expression was not associated with DFS and OS. Five-year DFS and OS rates were 13% and 43%, respectively, in patients with weak stromal CAV1 expression and 40% and 71%, respectively, in patients with strong stromal CAV1 expression. In this study, we indicate that weak stromal CAV1 expression in CRLM is an adverse prognostic factor in patients who undergo liver resection for liver-only colorectal metastases. We suggest validation of this finding in an independent cohort and consideration of risk stratification for post-hepatectomy adjuvant follow-up and therapy.

## Introduction

The treatment of colorectal liver metastases (CRLM) by surgical resection has become accepted and its use has increased over the last two decades. Surgical resection of CRLM has been shown to increase patient survival, with a five-year overall survival (OS) ranging between 35% and 58% for resected cases^1^. However, unfortunately, most of the patients who undergo liver resection will exhibit recurrence and only 16% of these patients are disease-free 10 years after hepatectomy^2^.

In an attempt to individualise perioperative systemic therapy, prognostic factors have been sought to classify hepatectomy patients at low and increased risk of disease recurrence^3–5^. Factors that have been demonstrated to have an effect on the prognosis of these patients include primary tumour stage and grade, interval from diagnosis of primary tumour to diagnosis of CRLM, the size of CRLM and its intrahepatic distribution, presence of extrahepatic disease, and response to neoadjuvant chemotherapy^3–5^.

Interaction between cancer cells and the stroma microenvironment has been identified as one of the main regulators of cancer development and progression^6^. The prognostic significance of a variety of stromal biomarkers in cancer patients has also been demonstrated^7^. Stromal expression of Caveolin-1, a protein of plasma membranes, is one of the most evaluated microenvironment biomarkers with prognostic significance^7–12^. The initial studies focussed on breast cancer, but more recently it became clear that the loss or the weak expression of CAV1 in the cancer stroma is correlated with adverse prognosis for a variety of cancers, such as gastric cancer, pancreatic adenocarcinoma, prostate cancer, and colorectal cancer^7–12^.

Loss of stromal CAV1 expression has been characterised as a key regulator in the development of the “reverse Warburg effect” and “the autophagic tumour stroma model of cancer metabolism”^13–15^. The autophagic tumour stroma model proposes that loss of stromal CAV1 expression, and more specifically the loss of CAV1 expression of cancer-associated fibroblasts (CAFs), is the result of the overproduction of reactive oxygen species (ROS) by the cancer cells^13–15^. Furthermore, CAFs with loss of CAV1 expression have an increased ability to provide nutrients to cancer cells, and in this fashion, promote aggressive tumour growth^13–15^.

The purpose of our study was to ascertain whether the stromal and cancer cell CAV1 expression in colorectal liver metastases affects the disease-free survival (DFS) and overall survival (OS) of patients with liver-only colorectal metastases undergoing hepatectomy.

## Materials and Methods

### Patients and Specimens

Patients who underwent liver resection for CRLM between January 2001 and December 2012 were identified from a prospectively collected First Department of Surgery, University of Athens Medical School, Laiko Teaching Hospital surgical database. Eligible patients were those who underwent liver resection for liver-only colorectal metastases following neoadjuvant chemotherapy for which histological and clinical parameters were available. Patients were excluded from the analysis if they had extrahepatic disease, did not receive neoadjuvant chemotherapy, underwent an incomplete resection (R2 resection), died in the postoperative period, or there was not enough formalin-fixed paraffin embedded tissue of good quality to evaluate the stromal and cancer cell CAV1 expression.

For the exclusion of extrahepatic disease, all patients underwent computed tomography of the chest, abdomen, and pelvis. The extent of liver disease in all patients was determined using magnetic resonance imaging (MRI). Resectability was evaluated by an interdisciplinary review that included a surgical expert, and all resections were initiated with a curative intent. Portal vein embolisation was performed 4 weeks prior to surgery in cases where the future liver remnant was considered inadequate (i.e., the ratio of the future liver remnant to the whole liver volume was <30%).

The institutional electronic records were checked for each patient and data was collected regarding (a) standard demographics, (b) primary colorectal tumour, (c) CRLM characteristics, (d) preoperative chemotherapy, (e) response to preoperative chemotherapy, (f) liver resection, and (g) DFS and OS.

The study was approved by the local ethics committee and the Institutional Review Board (Medical School, National and Kapodistrian University of Athens) and therefore performed in accordance with the ethical standards laid down in the Declaration of Helsinki. All patients sign informed consent for the immunochemistry of the specimens.

### Immunohistochemistry

Immunohistochemical staining for CAV1 was performed on 3-μm-thick formalin-fixed paraffin sections, using a two-step technique after overnight heating at 37 °C and subsequent deparaffinisation in xylene and rehydration through graded alcohols. After quenching of the endogenous peroxidase activity using a methanol hydrogen peroxide solution (0.3% in Tris-buffered saline [TBS] for 30 min), we proceeded to microwave-mediated antigen retrieval in ethylenediaminetetraacetic acid (EDTA) at pH 9.0 for 10 min. Subsequently, sections were incubated overnight at 4 °C with the primary antibody (CAV-1, clone sc-894, Santa Cruz Biotechnology Inc., USA). A two-step technique (Quanto, Thermo, Fischer Scientific Inc., USA) was used. Diaminobenzidine was used as a chromogen. Finally, sections were counterstained with hematoxylin and mounted.

As positive controls, we used lung cancer (CAV1) sections previously known to be highly immunoreactive for the studied markers. Negative controls had the primary antibody omitted and replaced by nonimmune, normal serum from the same species as the primary antibody or TBS.

### Evaluation of Immunostaining

The evaluation of the immunohistochemical staining was performed by two pathologists (G.A. and N.K) through light microscope observation. The pathologists were unaware of the clinical data of each patient.

Immunoreactivity for stromal CAV1 was estimated in a semiquantitative manner by the evaluation of staining intensity (score 0: no staining, score 1: weak staining, score 2: moderate staining, and score 3: strong staining) and the extent of positive cells over the total number of stromal cells (score 0: no staining, score 1: <25% of cells stained, score 2: 25–50% of cells stained, score 3: 50–75% of cells stained, score 4: >75% of tumour stained). The same semiquantitative method was used for the evaluation of cancer cell CAV1 expression.

The final immunoreactivity score was the sum of the staining intensity and the extent of positive cells. Any immunoreactivity score higher than 5 was considered to be strong CAV1 expression, while any immunoreactivity score equal to or less than 5 was considered to be weak CAV1 expression.

### Statistical Analysis

Statistical analyses were performed with the Statistical Package of the Social Sciences (SPSS) version 17.0. The primary endpoints of the study were DFS and OS. DFS was calculated from the date of hepatectomy to the date of disease recurrence and was censored at the last follow-up or at the time of death if the patients remained tumour-free at that time. OS was calculated from the time of hepatectomy to the date of cancer-related death and was censored at last follow-up or at the time of unrelated to cancer death.

The Chi-square test was used for calculating the association between patient and tumour categorical characteristics and level of stromal CAV1 expression. The impact of these features on DFS and OS was analysed using the Kaplan–Meier method. Survival outcomes between groups were compared with the log-rank test. A *P* value of less than 0.05 was considered statistically significant. The factors associated with the DFS or the OS (*P* > 0.1) in univariate analysis were used for the performance of the multivariate Cox regression analysis.

## Results

A total of 139 patients were identified from the institutional database; 109 patients were eligible for inclusion in the study. Patients were excluded for the following reasons: extrahepatic disease (*n* = 9), no neoadjuvant chemotherapy (*n* = 13 patients), incomplete resection/R2 resection (*n* = 3), postoperative death (*n* = 2), unavailable or bad-quality formalin-fixed paraffin-embedded tissue (*n* = 3).

The immunoexpression of CAV1, both membranous and cytoplasmic, was localised to stromal fibroblasts, cancer cells, and endothelial cells of blood vessels.

Patient demographics, characteristics of CRLM at diagnosis, details of surgical resection, and correlation of these characteristics with stromal CAV1 expression are shown in Table 1. The majority of patients were ≤70 years of age (82%) and most of them were male (62%). According to the Clinical Risk Score^5^, half of the patients (51%) belonged to the high-risk group. Only 43% of the patients had solitary liver metastases, while metastases were synchronous in 70% of patients. Sixty-five percent of patients were treated with oxaliplatin-based chemotherapy, and 40% were treated with preoperative bevacizumab. Only 9 patients (8%) experienced disease progression during preoperative chemotherapy.

**Table 1:** Relationships between baseline clinicopathologic characteristics and Stromal CAV1 expression.

Parameter	Total	Stromal CAV1 expression		P-value
		Weak	Strong	
**Age at operation**				
≤70 yr	89 (81.7%)	35 (74.5%)	54 (87.1%)	
>70 yr	20 (18.3%)	12 (25.5%)	8 (12.9%)	0.133
**Gender**				
Female	42 (38.5%)	20 (42.6%)	22 (35.5%)	
Male	67 (61.5%)	27 (57.4%)	40 (64.5%)	0.552
**Clinical Risk Score** ^**+**^				
Low Risk	55 (50.5%)	25 (53.2%)	30 (48.4%)	
High Risk	50 (45.8%)	21 (44.7%)	29 (46.8%)	
Unknown	4 (3.7%)	1 (2.1%)	3 (4.8%)	0.844
**No**. **of metastasis at diagnosis** ^**++**^				
1	47 (43.1%)	22 (46.8%)	25 (40.3%)	
>1	62 (56.9%)	25 (53.2%)	37 (59.7%)	0.560
**Size of largest metastases** ^**++**^				
≤5 cm	85 (78%)	36 (76.6%)	49 (79%)	
>5 cm	23 (21.1%)	11 (23.4%)	12 (19.4%)	
Unknown	1 (0.9%)	0 (0%)	1 (1.6%)	0.644
**Timing of metastasis** ^**++**^				
Synchronous	33 (30.3%)	16 (34%)	17 (27.4%)	
Metachronous	76 (69.7%)	31 (66%)	45 (72.6%)	0.530
**Preoperative CEA** ^**++**^				
≤200 ng/ml	104 (95.4%)	46 (97.9%)	58 (93.6%)	
>200 ng/ml	3 (2.8%)	1 (2.1%)	2 (3.2%)	
Unknown	2 (1.8%)	0 (0%)	2 (3.2%)	0.708
**Lymph node-positive primary tumour** ^**++**^				
No	29 (26.6%)	12 (25.5%)	17 (27.4%)	
Yes	77 (70.6%)	34 (72.4%)	43 (69.4%)	
Unknown	3 (2.8%)	1 (2.1%)	2 (3.2%)	0.830
**Type of Neoadjuvant chemotherapy**				
Oxaliplatin-based chemotherapy	71 (65.2%)	29 (61.7%)	42 (67.7%)	
Irinotecan- based chemotherapy	37 (33.9%)	18 (38.3%)	19 (30.7%)	
Other	1 (0.9%)	0 (0%)	1 (1.6%)	0.540
**Preoperative administration of Bevacizumab**				
No	66 (60.6%)	31 (66%)	35 (56.5%)	
Yes	43 (39.4%)	16 (34%)	27 (43.5%)	0.331
**Response to neoadjuvant chemotherapy** ^**+++**^				
Responders^+^	100 (91.7%)	43 (91.5%)	57 (91.9%)	
Progression	9 (8.3%)	4 (8.5%)	5 (8.1%)	0.933
**No**. **of segments removed**				
≤3	55 (50.5%)	23 (48.9%)	32 (51.6%)	
>3	54 (49.5%)	24 (51.1%)	30 (48.4%)	0.848
**Primary Tumour** ***in situ*** **at the time of hepatectomy**				
No	90 (82.6%)	39 (83%)	51 (82.3%)	
Yes^++++^	19 (17.4%)	8 (17%)	11 (17.7%)	0.999
**Adjuvant chemotherapy**				
Yes	82 (75.2%)	33 (70.2%)	49 (79%)	
No	27 (24.8%)	14 (29.8%)	13 (21%)	0.371
**Cancer cells CAV1 expression**				
Weak	38 (34.9%)	16 (34%)	22 (35.5%)	
Strong	70 (64.2%)	31 (66%)	39 (62.9%)	
Unknown	1 (0.9%)	0 (%)	1 (1.6%)	0.842

^+^Clinical Risk Score according to Fong Y *et al*.^5^.

^++^Clinical Variables included in the Clinical Risk Score^5^.

^+++^Radiologic Complete Response or Radiologic Partial Response or Stable Disease (according to RECIST)^16^.

^++++^15 patients underwent synchronous resection of primary tumour and CRLM and 4 patient were management with ‘liver first’ approach.

**Figure 1:**
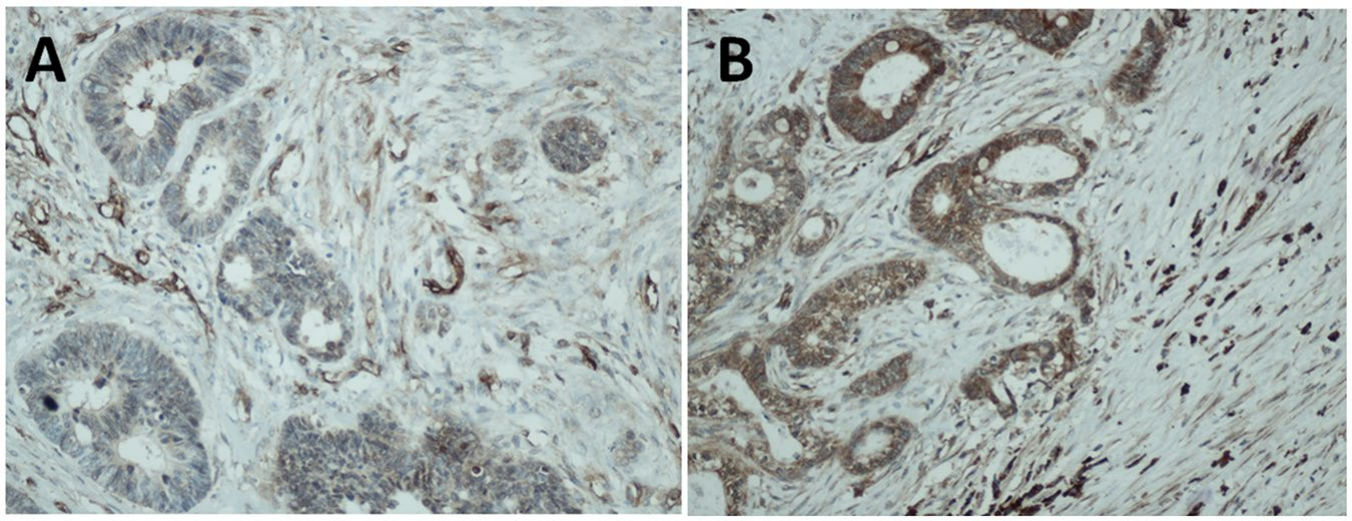
Immunohistochemical staining for caveolin-1. (**A**) Weak stromal caveolin-1 (CAV1) expression in colorectal liver metastases. (**B**) Strong stromal caveolin-1 (CAV1) expression in colorectal liver metastases.

Forty-seven patients (43%) demonstrated weak stromal CAV1 expression, and 38 patients (35%) demonstrated weak cancer cell CAV1 expression (Fig. 1). As shown in Table 1, there was no statistically significant difference in clinicopathologic characteristics between patients with weak and strong stromal CAV1 expression.

The median follow-up period for all patients was 39 months (2 to 99 months), while the median follow-up period for survivors was 45 months (2 to 99 months). During the follow-up period, 74 patients (68%) developed tumour recurrence and 39 (36%) patients died due to progressive disease. For the entire study population, the median DFS was 1 year, and the median OS was 6.8 years. Also for the entire study population, the 1-, 3-, and 5-year DFS rates were 52%, 34%, and 29%, respectively, whereas the 1-, 3- and 5-year OS rates were 96%, 75%, and 59%, respectively.

Within the group of patients with weak stromal CAV1 expression, the recurrence rate was much higher; 37 (79%) of the 47 patients developed tumour recurrence, giving weak stromal CAV1 expression a positive predictive value for recurrence of 79%. The corresponding rate for the group of patients with strong stromal CAV1 expression was 60% (79% vs. 60%, *P* = 0.040). Cancer-related death occurred in 24 of 47 (51%) patients with weak stromal CAV1 expression and in 15 of 62 (24%) patients with strong stromal CAV1 expression (*P* = 0.005).

**Figure 2:**
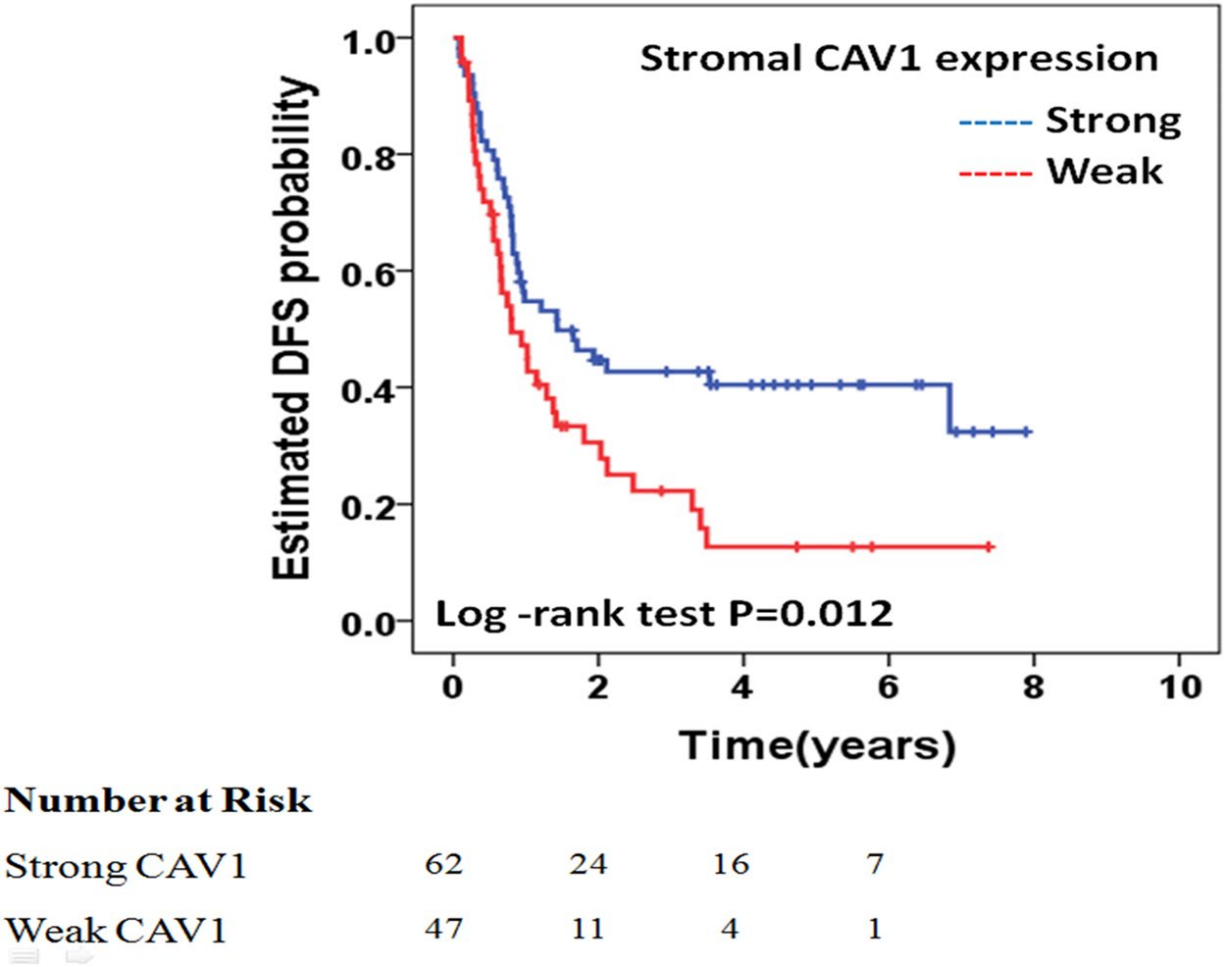
Kaplan–Meier analysis. Stromal CAV1 expression and disease-free survival. Weak stromal CAV1 expression is associated with a decreased DFS.

The results of univariate analyses (Table 2) demonstrated that high-risk patients according to the Clinical Risk Score^5^ (HR 1.96; 95% CI, 1.22–3.15; *P* = 0.005), disease progression during neoadjuvant chemotherapy according to the Response Evaluation Criteria in Solid Tumours (RECIST) criteria^16^ (HR 4.60; 95% CI, 2.20–9.60; *P* < 0.001), no administration of adjuvant post-hepatectomy chemotherapy (HR 2.30; 95% CI, 1.37–3.88; *P* = 0.002), and weak stromal CAV1 expression (HR 1.78; 95% CI, 1.13–2.83; *P* = 0.013) were associated with a decreased DFS (Fig. 2). There was no association between cancer cell CAV1 expression and DFS on univariate analyses (HR 1.01; 95% CI, 0.62–1.62; *P* = 0.966). Patients with weak stromal CAV1 expression had a median DFS of 9.6 months compared to a DFS of 17.2 months for patients with strong stromal CAV1 expression. Three- and 5-year DFS rates were 22% and 13%, respectively, in patients with weak stromal CAV1 expression and 42% and 40%, respectively, in patients with strong stromal CAV1 expression.

**Table 2:** Baseline clinicopathologic characteristics and their association with DFS and OS in univariate analysis and multivariate analysis.

Parameter	DFS				OS			
	Univariate analysis		Μultivariate analysis		Univariate analysis		Μultivariate analysis	
	HR(95% CI)	P- value	HR(95% CI)	P-value	HR(95% CI)	P-value	HR(95% CI)	P-value
**Age at operation**								
**≤**70 yr	1 (referent)				1 (referent)		1 (referent)	
>70 yr	1.11 (0.62–1.99)	0.718			2.12 (1.04–4.34)	**0**.**038**	1.65 (0.80–3.39)	0.173
**Gender**								
Female	1 (referent)				1 (referent)			
Male	1.51 (0.92–2.47)	0.101			1.34 (0.68–2.65)	0.394		
**Clinical Risk Score** ^+^								
Low Risk	1 (referent)		1 (referent)		1 (referent)		1 (referent)	
High Risk	1.96 (1.22–3.15)	**0**.**005**	1.88 (1.16–3.06)	**0**.**010**	2.31 (1.21–4.40)	**0**.**011**	2.11 (1.11–4.04)	**0**.**023**
**No**. **of metastasis at diagnosis** ^**++**^								
**1**	1 (referent)				1 (referent)			
>1	2.26 (1.38–3.71)	**0**.**001**			1.65 (0.86–3.17)	0.130		
**Size of largest metastases**								
**≤**5 cm	1 (referent)				1 (referent)			
>5 cm	1.20 (0.70–2.08)	0.493			1.48 (0.72–3.06)	0.279		
**Timing of metastasis**								
Synchronous	1 (referent)				1 (referent)			
Metachronous	1.42 (0.85–2.38)	0.179			1.37 (0.68–2.77)	0.372		
**Preoperative CEA** ^**++**^								
**≤**200 ng/ml	1 (referent)				1 (referent)			
>200 ng/ml	2.08 (0.65–6.65)	0.217			3.15 (0.95–10.4)	0.059		
**Lymph node-positive primary tumour** ^**++**^								
No	1 (referent)				1 (referent)			
Yes	2.33 (1.29–4.19)	**0**.**005**			2.85 (1.18–6.87)	**0**.**019**		
**Type of Neoadjuvant chemotherapy**								
Oxaliplatin-based chemotherapy	1 (referent)				1 (referent)			
Irinotecan- based chemotherapy	1.45 (0.90–2.33)	0.125			1.15 (0.58–2.28)	0.685		
**Preoperative administration of Bevacizumab**								
No	1 (referent)		1 (referent)		1 (referent)			
Yes	1.57 (0.98–2.49)	0.057	1.59 (0.97–2.60)	0.061	1.11 (0.58–2.13)	0.736		
**Response to neoadjuvant chemotherapy**								
Responders^+++^	1 (referent)		1 (referent)		1 (referent)			
Progression	4.60 (2.20–9.60)	**<0**.**001**	4.11 (1.90–8.89)	**<0**.**001**	1.75 (0.61–5.00)	0.289		
**No**. **of segments removed**								
**≤**3	1 (referent)				1 (referent)			
>3	1.35 (0.85–2.14)	0.197			1.11 (0.59–2.09)	0.729		
**Primary Tumour** ***in situ*** **at the time of hepatectomy**								
No	1 (referent)				1 (referent)			
Yes^++++^	1.35 (0.88–2.08)	0.169			1.06 (0.55–2.04)	0.842		
**Adjuvant chemotherapy**								
Yes	1 (referent)		1 (referent)		1 (referent)		1 (referent)	
No	2.30 (1.37–3.88)	**0**.**002**	1.89 (1.09–3.29)	**0**.**023**	2.94 (1.50–5.75)	**0**.**002**	2.35 (1.20–4.63)	**0**.**013**
**Stromal CAV1 expression**								
Strong	1 (referent)		1 (referent)		1 (referent)		1 (referent)	
Weak	1.78 (1.13–2.83)	**0**.**013**	2.00 (1.24–3.22)	**0**.**004**	2.82 (1.47–5.38)	**0**.**002**	2.47 (1.28–4.76)	**0**.**007**
**Cancer cells CAV1 expression**								
Strong	1 (referent)				1 (referent)			
Weak	1.01 (0.62–1.62)	0.966			1.14 (0.59–2.21)	0.681		

Abbreviation: DFS = disease free survival; OS = overall survival; HR = hazards ratio; CI = confidence interval;

^+^Clinical Risk Score according to Fong Y *et al*.^5^.

^++^Clinical Variables included in the Clinical Risk Score^5^.

^+++^Radiologic Complete Response or Radiologic Partial Response or Stable Disease (according to RECIST)^16^.

^++++^15 patients underwent synchronous resection of primary tumour and CRLM and 4 patient were management with ‘liver first’ approach.

**Figure 3:**
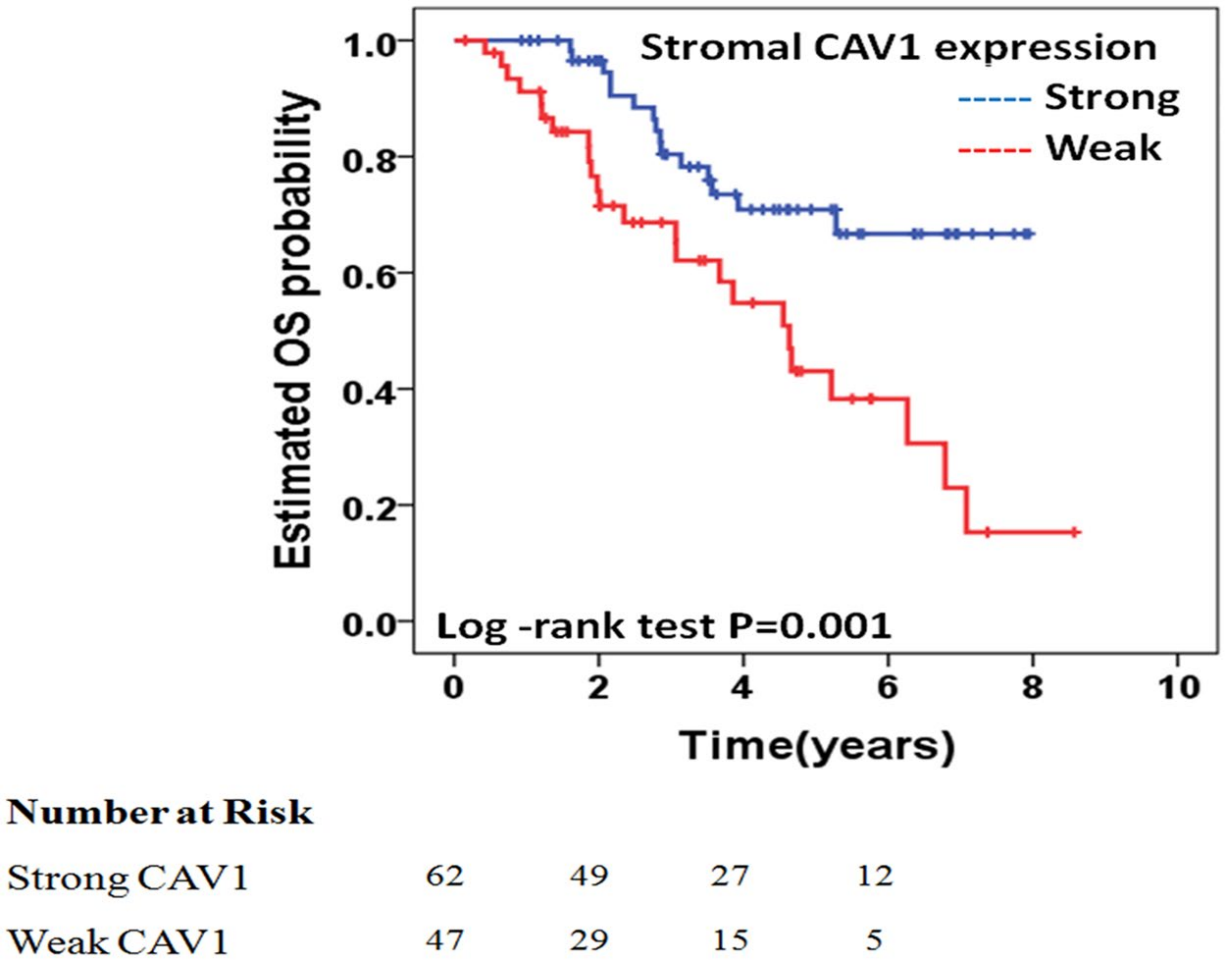
Kaplan–Meier analysis. Stromal CAV1 expression and overall survival. Weak stromal CAV1 expression is associated with a decreased OS.

Regarding OS, univariate analysis (Table 2) revealed that age greater than 70 years (HR 2.12; 95% CI, 1.04–4.34; *P* = 0.038), high-risk patients according to the Clinical Risk Score^5^ (HR 2.31; 95% CI, 1.21–4.40; *P* = 0.011), no administration of adjuvant post-hepatectomy chemotherapy (HR 2.94; 95% CI, 1.50–5.75; *P* = 0.002), and weak stromal CAV1 expression (HR 2.82; 95% CI, 1.47–5.38; *P* = 0.002) were associated with a decreased OS (Fig. 3). There was no association between cancer cell CAV1 expression and DFS on univariate analyses (HR 1.14; 95% CI, 0.59–2.21; *P* = 0.681). Patients with weak stromal CAV1 expression had a median OS of 56 months, while the median OS was not reached in patients with strong stromal CAV1 expression. Three- and 5-year OS rates were 69% and 43%, respectively, in patients with weak stromal CAV1 expression and 80% and 71%, respectively, in patients with strong stromal CAV1 expression.

Multivariate analysis for DFS was adjusted for risk of recurrence according to the Clinical Risk Score^5^, preoperative administration of bevacizumab, response to neoadjuvant chemotherapy, administration of adjuvant post-hepatectomy chemotherapy, and stromal CAV1 expression. For OS, the multivariate analysis was adjusted for age, risk of recurrence according to the Clinical Risk Score^5^, administration of adjuvant post-hepatectomy chemotherapy, and stromal CAV1 expression. In multivariate analysis, factors that remained statistically associated with DFS included high-risk patients according to the Clinical Risk Score^5^ (HR 1.88; 95% CI, 1.16–3.06; *P* = 0.010), disease progression during neoadjuvant chemotherapy according to the RECIST criteria^16^ (HR 4.11; 95% CI, 1.90–8.89; *P* < 0.001), no administration of adjuvant post-hepatectomy chemotherapy (HR 1.89; 95% CI, 1.09–3.29; *P* = 0.023), and weak stromal CAV1 expression (HR 2.00; 95% CI, 1.24–3.22; *P* = 0.004. Regarding OS, factors that remained statistically associated with OS in multivariate analysis were high risk patients according to the Clinical Risk Score^5^ (HR 2.11; 95% CI, 1.11–4.04; *P* = 0.023), no administration of adjuvant post-hepatectomy chemotherapy (HR 2.35; 95% CI, 1.20–4.63; *P* = 0.013), and weak stromal CAV1 expression (HR 2.47; 95% CI, 1.28–4.76; *P* = 0.007) (Table 2).

## Discussion

In our study, we demonstrate that in patients with liver-only colorectal metastases undergoing potentially curative hepatectomy, there is a significant association between stromal CAV1 expression and DFS and OS, but no correlation between cancer cell CAV1 expression and DFS and OS. A weak stromal CAV1 expression increases the likelihood of disease recurrence and cancer-related death, and decreases DFS and OS. Stromal CAV1 expression is not affected by the clinicopathological characteristics of the patients. Although the prognostic value of stromal CAV1 expression has been demonstrated in various types of cancer, to the best of our knowledge, this is the first study investigating the stromal CAV1 expression of metastases, and more specifically, in liver-only colorectal metastases^7–12^.

The most extensive research regarding the prognostic value of stromal CAV1 expression concerned breast cancer^8, 9, 17–21^. All these studies documented that loss of stromal CAV1 expression or weak expression is a regulatory key in all aspects of tumorigenesis and tumour progression. More specifically, it was shown that loss of stromal CAV1 expression accelerates the progression of ductal carcinoma *in situ* to invasive cancer and is associated with more advanced disease with higher T and N stages, higher recurrence rates, and decreased DFS and OS^8, 9, 17–21^. Interestingly, two of these studies showed that loss of stromal CAV1 expression is also associated with resistance to therapy with tamoxifen^9, 21^. Another interesting finding of these studies was that DFS and OS were associated with stromal CAV1 expression but not with cancer cell CAV1 expression^8, 9, 18^.

More recently, studies investigated the prognostic value of stromal CAV1 expression in various cancers, and more specifically in prostate cancer, gastric cancer, pancreatic cancer, non-small-cell lung cancer, and colorectal cancer^7, 10–12, 22^. All these studies concluded that loss of stromal CAV1 expression is associated with decreased DFS and OS. Furthermore, the results of all these studies confirm the findings of breast cancer studies that stromal CAV1 expression and not tumour cell CAV1 expression has of prognostic value^7, 10–12, 22^. In our study, we demonstrated for the first time that weak expression of CAV1 is associated with decreased DFS and OS in patients undergoing liver-resection for colorectal liver metastases.

Currently, it is accepted worldwide that tumorigenesis and tumour progression is not only associated with the transformation of cancer cells themselves, but is a complex process involving the crosstalk between cancer cells and the tumour microenvironment. The tumour microenvironment is consisted mainly of cancer-associated fibroblasts (CAFs), although it is not limited to CAFs. The study of CAFs is an emerging area of research as more and more studies find these cell functions as conducive to cancer growth, progression, and metastasis^23–26^. More specifically, it has been shown that CAFs accelerate the proliferation, survival, and migration of cancer cells, and prevent cancer cell apoptosis^23^. Also, the expression (or inexpression) of some markers by CAFs, including CAV1, has been shown to have prognostic value in various malignancies^7, 10–12, 22, 27^.

Although there is little knowledge regarding the origin of CAFs, loss of fibroblast CAV1 expression plays a crucial role in the phenotype transformation from benign fibroblasts to CAFs^28^. It has been shown that Caveolin-1−/− null mammary stromal fibroblasts and CAFs have a similar phenotype, and that this phenotype is reversed by treatment with a Cav-1 mimetic peptide^29, 30^. Although loss of stromal CAV1 expression may be attributed to various mechanisms (activation of oncogenes or inactivation of tumour suppression genes, activation of the TGF-β signalling pathway as it occurs in fibroblasts involved in wound healing), some recent studies demonstrated that cancer cells themselves accelerate the loss of CAV1 expression of stromal fibroblasts^31–34^.

Cancer cells secrete peroxide oxygen in the tumour microenvironment, inducing oxidative stress in surrounding fibroblasts^31^. Oxidative stress activates the HIF-1 and NF-kB transcription factors in stromal cells. This activation accelerates autophagy, mitophagy, and aerobic glycolysis in stromal fibroblasts. Autophagy, in turn, results in downregulation of CAV1 expression and phenotype transformation of benign fibroblasts into CAFs. Mitophagy, mitochondrial dysfunction, and aerobic glycolysis in stromal fibroblasts result in the production and secretion of lactate and ketones by the CAFs. These nutrients are recruited and used by cancer cells, which are characterised by normal if not increased mitochondrial function, and thus undergo oxidative mitochondrial metabolism. The sequence of events described above comprises the reverse Warburg effect and the autophagic tumour stroma model of cancer metabolism recently proposed by S. Pavlides *et al*.^35, 36^. Furthermore, some *in vivo* animal studies proposed that breaking this stromal–epithelial metabolic coupling may adversely affect the growth of cancer cells^15, 37^. It is unknown if breaking this coupling will have any effect on cancer treatment, and more studies investigating this possibility are needed.

## Conclusions

Our study, demonstrating a role for stromal CAV1 expression in disease-free and overall survival for patients with resected CRLM, adds to the growing body of evidence supporting the prognostic value of stromal CAV1 expression in perhaps all types of malignancies, and the utility of stromal CAV1 expression but not of cancer cell CAV1 expression for the classification of cancer patients into high- and low-risk groups for recurrence and cancer-related death. The greatest limitation of our study is its retrospective nature, and as such, selection bias is a possibility. The strength of our work includes a uniform approach to patient assessment, treatment, and surgery, and a resulting homogeneous patient population. We suggest validation of our findings in an independent cohort, with the aim of implementing more individualised treatments for patients undergoing hepatectomy for CRLM. In the future, further experience could also lead to the introduction of targeting therapies of either stromal CAV1 or other key regulators of the reverse Warburg effect and the autophagic tumour stroma model of cancer metabolism^15, 37^.

### Ethics approval and consent to participate

The study was approved by the local ethics committee and the Institutional Review Board and therefore performed in accordance with the ethical standards laid down in the Declaration of Helsinki.

### Consent for publication

All patients sign approval before operation for the immunochemistry of the specimens.
